# Disease recurrence in paediatric renal transplantation

**DOI:** 10.1007/s00467-009-1137-6

**Published:** 2009-02-27

**Authors:** Pierre Cochat, Sonia Fargue, Guillaume Mestrallet, Therese Jungraithmayr, Paulo Koch-Nogueira, Bruno Ranchin, Lothar Bernd Zimmerhackl

**Affiliations:** 1 Centre de référence des maladies rénales rares, Service de Pédiatrie & Inserm U820, Hôpital Femme Mère Enfant & Université de Lyon, 59 boulevard Pinel, 69677 Bron, France; 2 Medical Research Council Laboratory for Molecular Cell Biology, University College London, London, UK; 3 Department of Pediatrics, Medical University, Innsbruck, Austria; 4 Department of Pediatrics, Universidade Federal de São Paulo (UNIFESP), Escola Paulista de Medicina, São Paulo, Brazil

**Keywords:** Disease recurrence, Renal transplantation, Child, Focal and segmental glomerulosclerosis, Haemolytic uraemic syndrome, Membranoproliferative glomerulonephritis, Systemic lupus erythematosus, Primary hyperoxaluria type 1

## Abstract

Renal transplantation (Tx) is the treatment of choice for end-stage renal disease. The incidence of acute rejection after renal Tx has decreased because of improving early immunosuppression, but the risk of disease recurrence (DR) is becoming relatively high, with a greater prevalence in children than in adults, thereby increasing patient morbidity, graft loss (GL) and, sometimes, mortality rate. The current overall graft loss to DR is 7–8%, mainly due to primary glomerulonephritis (70–80%) and inherited metabolic diseases. The more typical presentation is a recurrence of the full disease, either with a high risk of GL (focal and segmental glomerulosclerosis 14–50% DR, 40–60% GL; atypical haemolytic uraemic syndrome 20–80% DR, 10–83% GL; membranoproliferative glomerulonephritis 30–100% DR, 17–61% GL; membranous nephropathy ∼30% DR, ∼50% GL; lipoprotein glomerulopathy ∼100% DR and GL; primary hyperoxaluria type 1 80–100% DR and GL) or with a low risk of GL [immunoglobulin (Ig)A nephropathy 36–60% DR, 7–10% GL; systemic lupus erythematosus 0–30% DR, 0–5% GL; anti-neutrophilic cytoplasmic antibody (ANCA)-associated glomerulonephritis]. Recurrence may also occur with a delayed risk of GL, such as insulin-dependent diabetes mellitus, sickle cell disease, endemic nephropathy, and sarcoidosis. In other primary diseases, the post-Tx course may be complicated by specific events that are different from overt recurrence: proteinuria or cancer in some genetic forms of nephrotic syndrome, anti-glomerular basement membrane antibodies-associated glomerulonephritis (Alport syndrome, Goodpasture syndrome), and graft involvement as a consequence of lower urinary tract abnormality or human immunodeficiency virus (HIV) nephropathy. Some other post-Tx conditions may mimic recurrence, such as de novo membranous glomerulonephritis, IgA nephropathy, microangiopathy, or isolated specific deposits (cystinosis, Fabry disease). Adequate strategies should therefore be added to kidney Tx, such as donor selection, associated liver Tx, plasmatherapy, specific immunosuppression protocols. In such conditions, very few patients may be excluded from kidney Tx only because of a major risk of DR and repeated GL. In the near future the issue of DR after kidney Tx may benefit from alternatives to organ Tx, such as recombinant proteins, specific monoclonal antibodies, cell/gene therapy, and chaperone molecules.

## Introduction

Renal transplantation (Tx) is the treatment of choice for stage 5 chronic kidney disease (CKD) and end-stage renal disease (ESRD). The risk of disease recurrence following renal Tx is becoming relatively high, due to increased immunosuppression and reduced acute rejection. Disease recurrence has a greater prevalence in children than in adults, thereby increasing patient morbidity, graft loss and, sometimes, mortality rates. Indeed, the current overall graft loss to acute rejection in children is 8–9% vs 7–8% to disease recurrence, mainly due to primary glomerulonephritis (70–80%) and inherited metabolic diseases (Table 1) [1–11]. Relative risk for graft failure in those with recurrent and de novo disease is 1.9 times that for the population in whom disease did not reoccur [3]. Changes in immunosuppression protocols that occurred during the past decades have not significantly modified the incidence of recurring diseases after Tx [3, 12].

**Table 1:** Recurrence of primary disease after the first renal transplantation [1–11] (*FSGS* focal segmental glomerulosclerosis, *HUS* haemolytic uraemic syndrome, *IgA* immunoglobulin A, *MPGN* membranoproliferative glomerulonephritis, *SLE* systemic lupus erythematosus)

Primary disease	Recurrence rate	Graft loss to recurrence
FSGS	14–50%	40–60%
Atypical HUS	20–80%	10–83%
Typical HUS	0–1%	0–1%
MPGN type 1	30–77%	17–50%
MPGN type 2	66–100%	25–61%
SLE nephritis	0–30%	0–5%
IgA nephritis (Berger disease)	35–60%	7–10%
Henoch–Schönlein nephritis	31–100%	8–22%
Primary hyperoxaluria type 1	90–100%	80–100%

The spectrum of recurrence is rather wide and mainly depends on the primary disease itself, ranging from the recurrence of the full disease—with various risks of graft loss—to the recurrence of specific features of the disease. In addition, some renal complications after transplantation may mimic recurrence but are based on different issues.

Unfortunately, there is limited evidence on the management of post-Tx disease recurrence, and clinical practice is usually based on non-randomized and uncontrolled case series.

## Recurrence of the ‘full’ disease

Recurrence with a high risk of graft loss

- Nephrotic syndrome

### Epidemiology

Of children with nephrotic syndrome (NS), 10–15% do not respond to corticosteroids and, among children with ESRD, 10–15% of them first present with the NS, i.e. the most common cause for renal Tx among acquired kidney diseases. Most of them show idiopathic focal and segmental glomerulosclerosis (FSGS) on renal biopsy examination, the yearly incidence of which seems to be increasing. The risk of recurrence in a first graft is 14–50%, with a risk of graft loss of 40–60%; in other words, the relative risk for graft failure from FSGS after Tx is 2.25 compared to non-recurrent diseases [1, 3, 4]. The risk of recurrence in a second graft after a first graft loss to recurrence is 60–100%. Some risk factors have been reported, as shown in Table 2. However, current knowledge on the pathophysiology of NS suggests that it may be either an immunological disease or a genetic disease, with a common histological pattern, i.e. FSGS, but has a different risk of recurrence (Table 3).

**Table 2:** Risk factors for recurrence of the nephrotic syndrome [1, 13, 14] (*HLA* human leukocyte antigen)

Proven increased risk	Independent/controversial risk factors	Proven decreased risk
Recurrence in a first graft	Gender	African–American recipients
Onset of NS during childhood	Mesangial hypercellularity	Genetic and syndromic NS
White and Asian recipients	Age at onset over 6 years	
Rapid course to ESRD (< 3 years)	Presence of FSGS circulating factor	
	Donor source	
	HLA typing/matching	
	Time interval on dialysis prior to Tx	
	Type of immunosuppressive therapy	
	Use of induction therapy	
	Bilateral nephrectomy of native kidneys

**Table 3:** Two groups of steroid-resistant nephrotic syndromes (*SRNS*) leading to end-stage renal disease in children (*GBM* glomerular basement membrane, *Sd* syndrome)

Primary idiopathic SRNS	Inherited SRNS	
*A multifactorial disease*	*A renal disease*	
T-cell dysregulation	Abnormal podocyte–GBM complex	
*Podocyte as a target*	*Podocyte as a disease*	
Idiopathic FSGS	*Genetic forms of NS*	*Syndromic forms of NS*
	Finnish type NS (*NPHS1*)	*WT1*-associated SRNS
	Other recessive FSGS	Pierson Sd, Schimke Sd
	(*NPHS2*, *NPHS3*)	Charcot–Marie–Tooth SRNS
	Dominant FSGS	
	(*ACTN4*, *TRPC6*, *CD2AP*)	
Circulating factor	No circulating factor	
Immunosuppression available	No effect of immunosuppression	

In some rare patients with the so-called ‘IgM nephropathy’ the disease has recurred after Tx, so that such form of steroid-resistant NS may be regarded as a distinct disease entity [15].

### Pathophysiology

The risk of recurrence mainly affects patients with primary idiopathic FSGS involving T-cell dysfunction and a putative circulating factor which may be bound to immunoglobulin. Several candidate agents have been proposed, such as cytokine, lymphokine, growth factor, and complement [16]. All of them may have the same endpoint, i.e. the glomerular epithelial cell.

### Genetics

Genetic underlying diseases have been described in FSGS. Recently, genes involved in the structure and function of the glomerular podocyte have been identified [17, 18]. The role of these genetic defects in the development of FSGS is still incompletely understood. However, in a recent series of 83 patients, those with a disease causing podocin mutation had no recurrence after renal Tx, whereas the risk of recurrence was over 50% in patients without mutation [19].

### Presentation

The recurrence of NS after Tx is based on FSGS as a primary disease, resulting in ESRD, presence of post-Tx nephrotic range proteinuria, with or without proven FSGS in the graft. The presentation of recurrence includes early massive proteinuria (78% during the first month after Tx, often as early as in the first urine from the graft) and sometimes graft failure and arterial hypertension [13]. There may be a delay in initial presentation coincident with acute tubular necrosis which occurs more frequently in renal Tx for FSGS. The onset of FSGS lesions is a secondary phenomenon which can occur early following recurrence of proteinuria.

### Prognosis

The prognosis of untreated recurrent NS is poor, and there is no current evidence of any kind of treatment. However, it is not ethical to keep the patient on dialysis, so that some therapeutic options have been advocated and include active treatments, preventive measures and rescue treatments [14].

### Global approach to transplantation

Some general measures are currently available: (1) an exact diagnosis is required in any patient; (2) DNA should be analysed/stored according to local convenience; (3) in patients with persistent NS, pre-emptive Tx may be dangerous because of the presence of heavy proteinuria from native kidneys, with the risk of both graft thrombosis and delayed diagnosis of recurrence; (4) pre-Tx bilateral nephrectomy is therefore recommended, with the patient on dialysis for a limited period of time. In addition, pre-Tx native nephrectomy may reduce the risk of recurrence per se; a reduction of the antigenic load that stimulates the putative permeability factor has been advocated [20].

Interestingly, the transplanted kidney provides a unique environment, since immunosuppressive agents [e.g. cyclosporine A (CsA)] that have not been successful on native kidneys may be effective in preventing post-Tx recurrence. Indeed, there is transient or no efficacy of such drugs on native kidneys because of their late use in the presence of advanced glomerular damage, whereas there is a potential benefit of early immunological intervention prior to Tx regarding the onset of glomerular damage in the graft.

In the absence of preventive measures, including pre-treatment of the recipient with CsA (with or without plasmapheresis), living donors should be avoided, since the relative risk of graft failure is 1.75 compared with that when kidneys from living donors are used in non-FSGS recipients [21]. This suggests a potential role for unknown genetic parameters that may trigger the development of FSGS.

### Experimental data

In rats CsA has been shown to prevent the increase in glomerular permeability caused by serum from patients with FSGS. On the other hand, other experiments in animal models have shown that glomerular permeability may be prevented by components of high-density lipoproteins, purified components of *Tripterygium wilfordii*, and 20-hydroxyeicosa-tetra-enoic acid (20-HETE).

### Active treatment of overt recurrence

Plasmapheresis using 4–5% albumin restitution (approximately ten sessions within 2 weeks, then weekly sessions for a total of 2 months, then adapted to individual response) has been successful in many patients. The use of more sophisticated methods, such as adsorption onto protein A columns or tryptophan immuno-adsorption, has no superior benefit [22]. Plasmapheresis has been used with or without cyclophosphamide instead of mycophenolate/azathioprine for an average of 12 weeks, on the basis of its long-lasting effect on T-cells [23]. Approximately 60% of patients attain complete remission, and the success rate is higher with early plasmapheresis, i.e. starting during the first 3–4 days after Tx. The adjunction of immunoglobulin replacement does not seem to have any benefit.

Controlled and randomized studies in children with primary SRNS have shown that CsA is effective in inducing remission. CsA may influence direct immunological phenomena and/or inhibit the effect of glomerular permeability factor; it may also act through vasoconstriction. High doses of CsA (either orally at a dose of 13–35 mg/kg per day, or with continuous infusion at a dose of 3 mg/kg per day in order to avoid nephrotoxic peak levels; blood concentration between 200 ng/ml and 350 ng/ml) for 1–3 weeks have given stimulating results in a significant number of patients [24, 25]. The rationale behind maintaining high CsA blood concentration is to overcome the effect of high serum levels of low-density lipoprotein (LDL) (secondary to recurrent NS), which binds CsA and is further responsible for low levels of free available CsA. However, some patients have required further plasmapheresis, and others have experienced CsA nephrotoxicity. In the long term, approximately 60% of the patients are in complete remission. The use of CsA as a first-step treatment may limit the efficacy of plasmapheresis, which may, therefore, be postponed.

The benefit of another anti-calcineurin agent, i.e. tacrolimus, on native kidney NS is scarce and has not been demonstrated in post-Tx recurrent NS so far; some patients experiencing a successful post-Tx strategy using CsA have experienced a late recurrence when switched from CsA to tacrolimus [14].

Antibody induction therapy using either anti-lymphocyte globulin or anti-interleukin (IL)2R antibodies (basiliximab, daclizumab) has given conflicting results, so that they are no longer used in such patients [24]. Recently rituximab, a chimeric monoclonal antibody that acts by inhibiting CD20-mediated B-cell proliferation and differentiation, has been used in some patients with recurrent NS under various conditions [timing of the first dose, associated post-transplantation lymphoproliferative disorder (PTLD)] [26]. Paediatric patients were given 375 mg/m^2^ × 1–6 without significant short-term side effect; of six cases reported in children, four went into complete remission [27–30] and two failed [31], so further data, including long-term outcomes, are expected. Comparable results have been published in adults [26].

### Preventive measures

The rationale of pre-treatment with CsA for 5–7 days (trough blood level 250–350 ng/ml) is based on experiment data. However, the use of a living donor is required for the planning of pre-treatment and further Tx. Encouraging results have been obtained from limited series (Fargue, in preparation). However, living donation should not be encouraged in the absence of such preventive measures.

### Rescue treatments

Angiotensin-converting enzyme (ACE) inhibitors, angiotensin 2-receptor blockers and non-steroidal anti-inflammatory drugs may partially reduce proteinuria without significantly affecting graft survival.

Many individual approaches have been proposed, especially for recurrence in a second graft. The risk of recurrence in such patients is incredibly high, and a strategy different from that for the first Tx must be suggested. Pre-treatment using both high doses of CsA and plasmapheresis should be considered [32]. In the post-Tx course any available option that was not attempted for the first graft may be proposed, such as high doses of CsA given intravenously, early plasmapheresis, cyclophosphamide, and rituximab.

- Haemolytic uraemic syndrome

Most children with haemolytic uraemic syndrome (HUS) present with a ‘typical’ form, with a 5–10% rate of ESRD, but 5–10% have an ‘atypical’ course (non-Shiga toxin-associated HUS), with a high risk of developing chronic kidney disease (CKD), so that HUS is the primary diagnosis in 2.5–5% of children with ESRD [5]. Indeed, more than 50% of patients with non-Shiga toxin-associated HUS progress to ESRD, and the overall outcome of kidney Tx is poor (Table 1). However, such atypical HUS is a group of distinct diseases that may be due to deficient von Willebrand factor-cleaving protease (ADAMTS 13), metabolic disorders, or various disorders of complement regulation [6, 8]. Complement abnormalities include specific gene mutations in factor H (20–30%), membrane cofactor protein (MCP, or CD46, 10–15%), factor I (10–15%), and factors B and C3; no gene mutation is found in 30–40% of the patients. In addition, approximately 5% of atypical HUSs are due to the presence of anti-factor H antibodies, leading to a secondary form of factor H deficiency. Patients with atypical HUS should, therefore, undergo complement factor determination (C3, factor H, factor I, factor B, and MCP expression), genotyping (factor H, factor H-linked genes, factor I, MCP, factor B and C3) and determination of anti-factor H antibodies prior to renal Tx so that the risk of graft failure can be evaluated [6].

Graft failure is often due to endothelial damage, i.e. vascular thrombosis and disease recurrence. Disease recurrence is approximately 0% to 1% in children with typical HUS and 20% to 80% in atypical HUS (Table 1) [5–7]. The average time to recurrence is around 1 month but can be late, after several months as well [8]. Recurrence may be triggered by viral or bacterial infection and by allograft rejection; it may also be increased in living donor Tx. Clinical symptoms of recurrence are often severe. The overall management and the risk of post-Tx recurrence are strongly linked to the underlying genotype and associated pathophysiology of HUS. CsA may cause de novo HUS in recipients of organ and bone marrow transplants, but it has no specific effect on outcome of primary HUS recurrence [33].

Patients with ADAMTS-13 deficiency present either with neonatal onset HUS or with a recurrent thrombotic thrombocytopenic purpura-like course. Few of them have received transplants, but recurrence is rather frequent and the patient can benefit from fresh frozen plasma infusion [34].

In MCP mutation, HUS usually presents in children older than 1 year of age as a recurrent disease of native kidneys. The transplanted kidney brings a normal MCP, so that the risk of recurrence after Tx is less than 20% [6, 35]. The option of using a living donor should not be ruled out when MCP mutation has been proven in the recipient and excluded in the donor.

In factors H, I or B mutations the clinical presentation is characterized by early symptoms, i.e. during the first 3 months of life. The recurrence rate in children with factor H deficiency is 66–76%, and most of them (77–93%) will lose their graft, so that living donors should be excluded [5, 6, 35]. Comparable poor results have been found in patients with factor I mutation: 88% recurrence rate with 100% graft loss after 1 year [6]. Very few patients with factor B or C3 mutation but no factor H deficiency have undergone Tx, but, again, the risk of recurrence seems to be rather high [5, 6]. The risk of recurrence both in the autosomal recessive and dominant forms of HUS of unknown mechanism is not documented in children, but it is approximately 60% in adults [5].

Factor H and factor I mutations are sometimes regarded as contraindications to kidney Tx. However, aggressive treatment with plasmapheresis using fresh frozen plasma (40–80 ml/kg per session) has provided interesting results in selected cases [6]. Patients with graft loss despite plasmapheresis require either long-term dialysis or another innovative therapeutic option. Human plasma-derived factor H concentrate has been suggested but is not yet available, whereas the recent use of anti-C5 monoclonal antibodies (eculizumab) may allow encouraging results. Because factor H is synthesized in the liver, combined liver and kidney Tx (together with pre- and intra-operative plasmapheresis using fresh frozen plasma and low molecular weight heparin) has been proposed for patients with severe forms of HUS, with variable results, the more recent being the more encouraging [6, 36, 37].

For patients with no identified mutation, there are two options: either wait until new genes are identified, or proceed with kidney Tx combined with intensive plasmatherapy [6].

Guidelines are accessible at http://www.espn.ucwm.ac.uk.

- Membranoproliferative glomerulonephritis

### Membranoproliferative glomerulonephritis type 1

Membranoproliferative glomerulonephritis type 1 (MPGN-1) accounts for approximately 2% of the primary diagnoses of children who receive renal transplants. The rate of recurrence in children has been reported to be between 30% and 77%, with graft failure to recurrence in 17– 50% [10, 38, 39]. Time to recurrence is 0.5–2 years, and the first sign of recurrence is proteinuria, which may lead to NS and further graft loss. Epstein–Barr virus infection may trigger the recurrence of glomerular disease [40]. There is no evidence of any successful treatment, despite anecdotal response to either plasmapheresis or long-term cyclophosphamide in adults [10, 41]; however, a combination of both strategies may be suggested.

### Membranoproliferative glomerulonephritis type 2

Membranoproliferative glomerulonephritis type 2 (MPGN-2) is an uncommon form of complement-dependent acquired renal disease that often leads to ESRD. It has been shown in adults that it recurs almost universally, and up to one-quarter of patients lose their graft from recurrence [40]. A retrospective analysis of 75 children found that the 5-year graft survival rate in MPGN-2 was 50.0 ± 7.5%, compared with 74.3 ± 0.6% in patients with other primary diseases (*P* < 0.001) [42]. Organs from living related donors have a better 5-year survival rate than do those from deceased donors (65.9 ± 10.7% and 34.1 ± 9.8%, respectively, *P* = 0.004). Disease recurrence was present in two-thirds of the 18 patients who underwent transplant biopsy and was responsible for all 11 graft losses. The risk of recurrence and graft loss is independent of both pre-Tx presentation and C3 concentration, but it is strongly associated with the presence of heavy proteinuria, which is the hallmark of clinical recurrence. The presence of glomerular crescents was negatively correlated with graft survival, and patients with histological recurrence experienced higher serum creatinine concentration and urine protein excretion. In addition, the risk of graft loss seems to be lower in adults than in children. There is no available treatment for MPGN-2 recurrence.

Recurrence has not been reported in the very rare patients with lipodystrophy (Barraquer–Simons disease) -associated MPGN-2.

- Membranous nephropathy

In adults the risk of post-Tx recurrence of membranous nephropathy reaches 29% after 3 years, without evidence of specific risk factors; such a recurrence is associated with a high risk of graft loss, i.e. 38% and 52% after 5 years and 10 years, respectively [43].

- Lipoprotein glomerulopathy

ESRD due to lipoprotein glomerulopathy is an exceptionally rare condition in children. In adults the risk of post-Tx recurrence approximates 100% after an average time interval of 7 months, and the risk of graft loss to recurrence involves most patients [44]. Neither specific treatment nor prevention has been advocated, except lipid lowering and ACE inhibitor therapy. The use of living donors, therefore, cannot be recommended.

- Primary hyperoxaluria type 1

Primary hyperoxaluria type 1 (PH-1) is a recessive autosomal disease caused by a deficiency of hepatic peroxisomal alanine:glyoxylate aminotransferase (AGT; cofactor: pyridoxal phosphate), which catalyses the conversion of glyoxylate to glycine. This leads to overproduction of oxalate and massive urinary excretion of monohydrated calcium oxalate. Once CKD has been reached due to progressive urolithiasis and nephrocalcinosis, insoluble oxalates accumulate throughout the body and mainly skeleton and vessels [45]. Since the metabolic defect is in the liver, isolated kidney Tx cannot correct the primary metabolic defect but only the damaged target organ. Indeed, the risk of recurrence in the absence of liver Tx is 90–100%. A combined liver and kidney Tx is therefore required for most patients. However, in patients with a long period of ESRD, the oxalate release from the body may jeopardize the renal graft, with a picture of recurrence, despite the metabolic correction associated with synchronous liver Tx. This is the reason why patients with PH-1 should undergo transplantation pre-emptively when their glomerular filtration rate (GFR) approximates 30–20 ml/min per 1.73 m^2^. Some patients who receive a transplant after a long period of time on dialysis may benefit from a sequential liver and kidney Tx so that systemic oxalate may be cleared by dialysis between the liver and kidney Tx procedures [45].

For any patient, the diagnosis has to be confirmed from DNA analysis (or from AGT activity measured from a liver biopsy) before any procedure of organ Tx is considered. Indeed, some patients may present with hyperoxaluria without AGT deficiency, leading to a diagnosis of either PH-2 (glyoxylate reductase/hydroxypyruvate reductase deficiency) or non-PH-1 non-PH-2 where Tx strategy has not been clearly delineated. DNA analysis for patients with PH-1 is of pivotal interest, since (1) a small number of patients with the Gly170Arg *AGXT* mutation (Western Europe) may present with evidence of pyridoxine responsiveness, sometimes allowing isolated kidney Tx with lifelong pyridoxine intake and (2) patients with the Ile244Thr *AGXT* mutation (North Africa, Spain) do not respond to pyridoxine and, therefore, require combined liver and kidney Tx; (3) experience of other mutations is limited and leads to the recommendation of combined liver and kidney Tx [45–47]. However, correlation with clinical phenotype and treatment response is complicated by the involvement of other genetic (e.g. modifier genes) and non-genetic (e.g. environmental) factors that affect disease severity [48].

Recurrence with a low risk of graft loss

- IgA nephropathy

### Berger disease

Up to 25% of patients with IgA nephropathy develop ESRD, and 35–60% will experience a histological recurrence of the disease [11, 49, 50]. These patients present with persistent microscopic haematuria and proteinuria, and renal transplant biopsy often shows mesangioproliferative glomerulonephritis, and not only silent recurrent mesangial IgA deposits from protocol biopsy. The risk of recurrence is not correlated with donor status, recipient age, race, gender, or immunosuppression [51]. After an average follow-up period of 61 months, 18 of 63 adult patients experienced recurrence, which led to graft loss in six [50]. Younger adult patients seem to be more prone to the risk of recurrence, but recurrence rate in children has not been documented enough; however, the percentage of graft loss to recurrence is approximately 7% [11, 52]. Proteinuria is associated with progressive loss of function in all patients with recurrent IgA nephropathy.

### Henoch–Schönlein purpura

The recurrence rate of Henoch–Schönlein purpura (HSP) after transplantation in children is common from protocol biopsies, but most data come from series of adults. There is an increased risk of disease recurrence in the aggressive progression to ESRD and when a living related donor has been used, but there is no influence from the type of post-transplantation immunosuppression [53, 54].

- Systemic lupus erythematosus

Results of kidney Tx in systemic lupus erythematosus (SLE) are rather good, since there is no significant risk of clinical recurrence [9, 55]. Some series report a 30% histological rejection rate and a greater risk of thrombotic complications (particularly in patients with antiphospholipid antibody syndrome) [9, 56]. Such an overall favourable outcome is probably due to the adequate immunosuppressive effect of anti-rejection regimens that keeps SLE in remission. In adults the overall graft survival rate is 87% after 1 year and 60% after 5 years [56]. In children SLE is responsible for approximately 3% of ESRD leading to kidney Tx in North America; allograft survival is not different from that in non-lupus renal diseases, but patient survival is worse, i.e. 1.8 relative risk of death in multivariable analysis [57].

- Anti-neutrophilic cytoplasmic antibody-associated small vessels vasculitides

Recurrence of idiopathic, pauci-immune, anti-neutrophilic, cytoplasmic antibody (ANCA)-associated (usually antimyeloperoxidase) necrotizing glomerulonephritis after renal Tx is rare, thanks to the adequacy of immunosuppression, so that the overall results are good. A combination of cyclophosphamide, corticosteroids and, sometimes, plasmapheresis has been successful in case reports [58, 59]. Interestingly, sirolimus may lower ANCA titres before kidney Tx and, therefore, may be considered in the post-Tx immunosuppression regime [60].

Wegener granulomatosis is a very rare cause of graft loss in children. The experience of renal Tx in adults shows that the risk of graft loss to recurrence ranges between 0% and 6%, with a comparable outcome to that in other non-diabetic primary diseases [55, 61].

- Methylmalonic acidaemia

Kidney Tx has provided acceptable results for the small number of patients with ESRD due to methylmalonic acidaemia (MMA). However, systemic production of MMA persists after kidney Tx, despite dietary protein restriction, so that plasma MMA remains at a lower range than before Tx according to post-Tx GFR but still many times greater than that in healthy subjects [62]. The progression of the disease after Tx therefore depends on both compliance and probably other individual metabolic parameters such as genotype, so that combined liver and kidney Tx has been proposed for selected patients, with variable results.

## Recurrence with a long-term risk of graft loss

### Insulin-dependent diabetes mellitus, sickle cell disease, endemic/Balkan nephropathy

Some renal diseases may lead to ESRD during late childhood or early adulthood, with a high risk of recurrence for as long as the primary disease causing the disorder is present. Such a recurrence is a delayed phenomenon and always occurs in adulthood, many years after the primary renal Tx. This has been shown for inherited diseases (e.g. insulin-dependent diabetes mellitus, sickle cell disease) as well as for some acquired diseases (e.g. endemic/Balkan nephropathy) when the causing agent is still present [63].

### Sarcoidosis

Renal involvement leading to CKD and further ESRD is rare, mainly in children. Very few cases of sarcoidosis recurrence in renal allograft recipients have been published, all of them affecting adults [64].

### Adenine phosphoribosyl-transferase deficiency

Very few patients with adenine phosphoribosyl-transferase deficiency and 2,8-dihydroxyadenine tubulointerstitial lesions present with ESRD of ‘unknown origin’ and no history of nephrolithiasis. Some of them, therefore, undergo transplantation in adulthood, with a risk of post-Tx stone recurrence [65]. Once the diagnosis has finally been established, a treatment based on allopurinol, purine-restricted diet and hydration is effective.

## Recurrence/occurrence of specific features of the primary disease

A large number of hereditary renal diseases may lead to ESRD and kidney Tx. In general, due to the molecular pathophysiology of such diseases, there is no risk of recurrence, except for some patients in whom graft function may be jeopardized by the occurrence of specific auto-antibodies.

### Genetic forms of NS

- Proteinuria

Owing to constitutional glomerular abnormalities, there is no risk of recurrence of the primary disease in inherited NS (Table 2) after Tx of a ‘new kidney’ [14, 17]. This has been shown for most patients with dominant FSGS and for patients with syndromic NS (*WT1*-associated NS, Schimke disease, Pierson syndrome, Galloway syndrome, Charcot–Marie–Tooth disease).

However, some patients with genetic NS present with proteinuria after Tx in the absence of specific features of the primary disease; this may be due to antibody-mediated glomerular injury leading to increased permeability and proteinuria. Such a complication has been found in patients with the Finnish type NS (anti-nephrin antibodies in *Maj/Maj* homozygous patients) and recessive FSGS (anti-podocin antibodies).

- Cancer

Patients with *WT1* mutations have an increased intrinsic risk of cancer in residual tissues, so that target organs must be removed prior to Tx, i.e. bilateral nephrectomy in Denys–Drash syndrome and bilateral gonadectomy in Frasier syndrome. In the case of tumours, prior to Tx, a one to two-year interval between the last specific treatment course and Tx is recommended [66].

- Other organ involvements

Very few patients present with Schimke immuno-osseous dysplasia, including FSGS-related CKD, combined immune deficiency, spondylo-epiphyseal dysplasia, vascular endothelial disease and specific morphological abnormalities. Most of these patients die during the first years of life because of severe opportunistic infections, but some of them survive further ESRD. In such recipients, there is no increased risk of post-Tx recurrent proteinuria or NS, but kidney Tx is still a life-threatening option because of additional immunosuppression. Interestingly, some patients have been successfully treated with kidney Tx following prior bone marrow Tx [67].

### Anti-glomerular basement membrane antibodies-associated glomerulonephritis

- Alport syndrome

Anti-glomerular basement membrane GBM) antibodies occasionally occur in Alport patients after renal allograft Tx. The association of high titres anti-GBM antibodies and vascular rejection may be important so that anti-GBM antibodies should be sought in the presence of vascular rejection in Alport patients [68].

- Goodpasture syndrome

Goodpasture syndrome is a rare cause of ESRD in children. Graft loss to recurrence has been estimated to be approximately 14% in adults, but the recurrence of anti-GBM nephritis has rarely been documented, either in the native kidneys or after renal Tx [55]. However, some patients with recurrent fulminant anti-GBM nephritis have been reported after Tx with the reappearance of circulating anti-GBM antibodies at the time of recurrence [69].

### Graft involvement as a consequence of lower urinary tract abnormality

A normal bladder acts as a low pressure, good volume, urinary reservoir that is continent, sterile and empties freely and completely. When such an environment is not achieved, infection and renal impairment may occur and recur after kidney Tx. Abnormal bladders must, therefore, be assessed urodynamically prior to Tx. A multidisciplinary team approach is required for such patients.

In most series of adults, as well as in children, patients with abnormal bladders, mainly posterior urethral valves, or urinary diversion experience worse graft function than those do with primary renal diseases, but graft survival is comparable [70]. Graft dysfunction may sometimes be due to both repeated urinary tract infection and abnormal urinary pressure, so limited surgical interventions prior to Tx and post-Tx antibiotic prophylaxis are recommended [71].

### Human immunodeficiency virus infection

Human immunodeficiency virus (HIV) nephropathy may lead to ESRD and involves 7–12% of African–American HIV-infected male patients [72]. Thanks to current highly active anti-retroviral therapy, HIV patients are now exposed to the risk of suffering from ESRD, and renal Tx may be proposed for selected paediatric recipients [73]. Indeed, there are now several reports of favourable outcome after Tx, since immunosuppression may have beneficial effects for such patients through moderation of immune activation or reduction of HIV reservoirs. Specific immunosuppressants drugs also have antiviral properties or interact positively with certain antiviral agents [72].

### Thrombosis

Patients with antiphospholipid antibody syndrome are at high risk of post-Tx renal thrombosis that could be prevented by anticoagulation therapy [74].

Inherited thrombophilia may lead to venous thrombosis, microvascular occlusion or acute rejection, with major consequences for graft survival. Some specific genotypes, mainly prothrombin mutations, are associated with such causes of graft failure, so pre-Tx screening for thrombophilia is recommended and should lead to pre- and post-Tx anticoagulation strategies [75].

## Different from recurrence

### De novo diseases

- Focal segmental glomerulosclerosis

The occurrence of late de novo FSGS after Tx may be due to chronic transplant nephropathy, anticalcineurin toxicity, etc.

- Thrombotic microangiopathy

Thrombotic microangiopathy (TMA) is characterized by aggregation of platelets in the renal and/or systemic circulation, thrombocytopenia and intravascular haemolysis. Independent of a recurrence of the original disease, it has been reported after Tx in 0.8% of patients in the United States of America, mainly during the first 3 months after Tx. Risk factors for the whole Tx population include younger recipient age, older donor age, female recipient, initial use of sirolimus and calcineurin inhibitors, acute rejection and viral infection [76, 77]. Of adults treated with calcineurin inhibitors, 3% develop de novo TMA; 62% present with systemic TMA and 38% with TMA limited to the graft; the prognosis is better in patients with localized forms who show a better response to reduction, conversion or temporary discontinuation of calcineurin-inhibitor treatment.

- IgA nephropathy

De novo mesangial deposition of IgA in renal allografts is not a rare feature and is usually clinically benign. However, some case reports have shown that it can present with crescentic lesions and lead to deterioration of graft function and, sometimes, graft loss [78].

- Diabetic nephropathy

De novo diabetic nephropathy is a rare event after Tx but may follow a decade-long course and further lead to graft loss [63].

### Isolated specific deposits

- Cystinosis

Renal transplantation has significantly prolonged the survival of patients with infantile cystinosis, so that the long-term outcome of the disease has changed during the past decades. Indeed, cystine continues to accumulate unabated in most organs and will lead to late systemic complications, such as visual impairment, hypothyroidism, central nervous system involvement, distal myopathy, and endocrine pancreatic insufficiency, which may trigger post-Tx diabetes mellitus at the time of glucocorticoid-based immunosuppression [79]. Most risks may be postponed by the daily use of cysteamine as soon as the diagnosis has been made, as well as following kidney Tx and later on.

There is no risk of recurrence of the primary disease in the graft, but protocol biopsies have shown that cystine crystals may be deposited within the interstitial tissue without any clinical or biological manifestation.

- Fabry disease

Fabry-related ESRD occurs in adulthood, but the challenge of kidney Tx in these patients must be known as early as when the diagnosis has been confirmed, i.e. in children and adolescents. Transplants in adults have an excellent graft survival rate, which is statistically similar to the rates of graft survival in patients without Fabry disease [80]. Surprisingly, from the North American experience in adults, patient survival rate and the risk of cardiovascular death in untreated patients with Fabry disease is comparable to those of other renal Tx recipients [80]. Such results may be due to the selection of patients, and there is now a rationale to treat any kidney transplant patient with enzyme replacement therapy, as renal Tx does not correct the underlying metabolic defect in other organs [81].

### Infection

Independent of primary disease, some patients lose their graft from polyoma BK virus-acquired nephropathy. Re-transplantation may be successful in the absence of graft nephrectomy, provided that findings for BK virus DNA were negative, both in the blood and in the urine (or kidney material) at the time of re-transplantation [82].

## Conclusions

Disease recurrence is currently one of the most challenging issue in paediatric kidney transplantation. However, graft loss and patient morbidity may benefit from a better approach to disease pathophysiology, better risk evaluation and improved individualized treatment strategies. DNA analysis should be performed as far as available, but adequate material should be stored for further investigations. Specific strategies should be added to kidney transplantation if necessary, such as donor selection, associated liver transplantation, plasmapheresis, and specific immunosuppression protocols. Therefore, very few patients may be excluded from kidney transplantation only because of a major risk of disease recurrence and repeated graft loss. In the near future, the issue of disease recurrence after kidney transplantation may benefit from new approaches to alternatives to organ transplantation, such as recombinant proteins, specific monoclonal antibodies, cell therapy, gene therapy, and chaperone molecules. The use of international registries and databases is of major concern in any project including interventional study.
